# Medicinal Preparations and Substitutes

**Published:** 1918-12-21

**Authors:** 


### MEDICINAL PREPARATIONS AND SUBSTITUTES.

The term "substitute" in relation to articles of food or medicine carries a decidedly unpleasant atmosphere. The vendor may affirm that it is "just as good" as the genuine article, but previous experiences of a similar order invite disbelief rather than confidence. If we may judge from the newspapers, the invention and manufacture of "substitutes" has been cultivated with much ingenuity in enemy countries, but the most recent comments do not show that these have redeemed the reproach which belongs generally to their tribe. They have not been cheap, it is true, but, on the other hand, they have not failed to be nasty. At home we have suffered but little from these dubious contrivances, and the only examples in which an organised presentation of them has occurred are found in certain medicinal preparations. Sugar, glycerine, fats and oils have been in short supply in consequence of the war, and even the pharmacy and the dispensing department have been raided in order to meet military demands. Munitions, at least for the time being, were more important than syrups and gargles, and patients have had less pleasant medicines in order that gunners may have more effective shells. Fortunately legal arrangements rendered it possible for the General Medical Council to authorise dispensers to use alternative preparations when medicines containing the forbidden articles were prescribed, and in this way difficulties have been surmounted or avoided. There is no need to be too severe on the permitted "substitutes," but it may be hoped that their temporary service will soon be declared unnecessary. They have served a purpose of some value. But we trust their day is nearly done, and that the authorities as soon as possible will restore the original pharmacopœial preparations.

A recent comment by a medical speaker suggested that the number of drugs and preparations recognised by the Pharmacopœia could well be reduced by at least 50 per cent. The criticism has a certain attractive quality, and more than one medical reformer has advanced it. Yet it rests, we venture to say, upon a complete misconception of the motive and purpose of a National Pharmacopœia. The object of such a volume is not to meet the convenience of the medical profession, but to serve the interests of the public. What the Legislature has aimed at is the creation of a standard which will ensure that the name of any medicine in more or less general use shall have one and the same quantitative and qualitative meaning in all parts of the country—so that the public may be protected from the possible vagaries of individual dispensers. This is secured by the definitions of the Pharmacopœia. Clearly, then, if the volume is to fulfil its main purpose it must contain the names of all medicines in more or less general use. Whether all these have medicinal value is a question on which opinions differ, and the debate on such an issue may engage the doctors at their will. The Pharmacopœia has no title to settle the point, and does not pretend to do so; it is not a text-book either of pharmacology or of therapeutics. But it is an authoritative standard of interpretation of the meaning of medicinal terms, and it is in the interests of the public that it should include all such terms as are, in fact, more or less commonly employed. To exclude any of these terms would be to defeat the end which the Pharmacopœia is created to serve.

